# Determinants of MoCA improvement in post-ischemic stroke patients receiving acupuncture: a retrospective study

**DOI:** 10.3389/fneur.2026.1892576

**Published:** 2026-10-27

**Authors:** Hui Long, Pengliang Wu

**Affiliations:** Encephalopathy Department, Shaanxi Provincial Hospital of Traditional Chinese Medicine, Xi'an, Shaanxi, China

**Keywords:** acupuncture, cognitive, cognitive impairment, retrospective study, stroke

## Abstract

**Background:**

Acupuncture is commonly integrated into multimodal rehabilitation programs for post-ischemic stroke cognitive impairment (PSCI), but the factors associated with cognitive improvement in this complex treatment context remain unclear. This retrospective study investigated factors associated with cognitive improvement in stroke patients receiving acupuncture as part of a standardized multimodal inpatient rehabilitation program.

**Methods:**

In this single-center retrospective cohort study, 226 ischemic stroke patients treated with acupuncture between January 2023 and December 2025 were enrolled. Demographic data, clinical characteristics, acupuncture treatment parameters, and laboratory indicators were collected from electronic medical records. Cognitive function was assessed using MoCA, MMSE, LOTCA, MBI, HAMD, HAMA, and PSQI scales. Patients were categorized into improvement (ΔMoCA ≥2) and non-improvement groups. Univariate and multivariate logistic regression analyses were used to identify factors associated with cognitive improvement.

**Results:**

Among the 226 patients, 148 (65.49%) achieved improvement. After treatment, the improvement group showed significant improvements in MoCA and MMSE scores compared with baseline and the non-improvement group [between-group difference for post-treatment MoCA: 1.59 (95% CI 0.82 to 2.36; *p* < 0.001); MMSE: 2.44 (95% CI 1.69 to 3.19; *p* < 0.001)]. Significant improvements were also observed in LOTCA, MBI, HAMD, HAMA, and PSQI scores in the improvement group (*p* < 0.001). Univariate analysis identified age, education years, admission NIHSS score, diabetes mellitus, time to acupuncture intervention, treatment frequency, and total treatment course as factors associated with recovery (*p* < 0.05). Multivariate analysis demonstrated that advanced age (OR = 1.208, *p* < 0.001), higher NIHSS score (OR = 1.643, *p* = 0.001), longer time from stroke onset to acupuncture intervention (OR = 1.162, *p* = 0.011) and diabetes mellitus (OR = 4.737, *p* = 0.018) were associated with a lower probability of achieving the ΔMoCA improvement threshold. In contrast, longer education duration (OR = 0.740, *p* = 0.006), higher treatment frequency (OR = 0.271, *p* < 0.001), and longer treatment course (OR = 0.248, *p* < 0.001) were associated with a higher probability of meeting the improvement threshold. However, as treatment frequency and total course were determined during the clinical course rather than at baseline, these associations should be interpreted as exploratory and hypothesis-generating, and do not imply causality.

**Conclusion:**

In this retrospective cohort of PSCI patients receiving acupuncture within a multimodal inpatient rehabilitation setting, shorter time from stroke onset to acupuncture initiation, higher weekly frequency, and longer treatment duration were exploratory factors associated with a greater likelihood of meeting the predefined ΔMoCA improvement threshold in this cohort. These hypothesis-generating observations may inform the design of future prospective studies and suggest patient characteristics that warrant further investigation. However, given the uncontrolled design, these findings cannot establish acupuncture-specific efficacy and should be considered hypothesis-generating, warranting confirmation through prospective randomized controlled trials.

## 1 Introduction

Stroke is a condition caused by cerebrovascular diseases that leads to impairment of brain function (1). After onset, patients typically experience motor and sensory dysfunction on one side of the body, and some patients may develop varying degrees of cognitive impairment. Cognitive dysfunction primarily manifests as deficits in memory, language ability, executive function, judgment, and calculation ability (2). Specific presentations include decline in recent and short-term memory, verbatim repetition, incoherent speech, slow speech, difficulty making choices, and reduced calculation ability (3). Post-stroke cognitive impairment (PSCI) is classified into mild, moderate, and severe grades based on the severity of cognitive dysfunction (4). PSCI has gradually become a significant contributor to the post-stroke disease burden, as it reduces the patient’s comprehension and cooperation during rehabilitation training, hinders the rehabilitation process, leads to decreased quality of life, and shortens patient survival time (5).

Worldwide, stroke is the second leading cause of death, with about two-thirds of these fatalities occurring in developing countries (6, 7). According to the Global Stroke Fact Sheet 2025 released by the World Stroke Organization (WSO), there are approximately 11.94 million new stroke cases annually worldwide, with about 93.81 million stroke survivors. The annual number of stroke-related deaths exceeds 7.25 million, resulting in a substantial loss of disability-adjusted life years (DALYs) (8). Currently, the prevalence of stroke in China ranks highest in the world (9, 10).

Despite the availability of conventional pharmacological and rehabilitative interventions for PSCI, their clinical efficacy remains suboptimal and highly variable. Current pharmacological options, such as cholinesterase inhibitors and memantine, have shown only modest and inconsistent benefits in post-stroke populations, often accompanied by adverse effects that limit their long-term use (11). Similarly, standard cognitive rehabilitation programs face challenges including low patient adherence, high resource intensity, and a lack of standardized protocols tailored to individual patient profiles (12). These limitations underscore a significant unmet clinical need: identifying modifiable therapeutic factors and patient characteristics that can guide more effective, individualized rehabilitation strategies. This gap in knowledge forms the primary rationale for investigating factors associated with cognitive improvement in PSCI patients, particularly in the context of acupuncture therapy.

Acupuncture, a widely used non-pharmacological intervention, is recommended by the World Health Organization (WHO) as an alternative and complementary strategy for improving stroke care (13). Acupuncture has a long history and extensive clinical experience in treating PSCI, and its efficacy has been preliminarily validated in numerous clinical studies. In recent years, multiple studies have shown that acupuncture can significantly improve cognitive function in post-stroke patients, increase scores on the MoCA and the MMSE, and also demonstrate positive effects on improving activities of daily living (14, 15). Although significant progress has been made in research on the efficacy and mechanisms of acupuncture for PSCI, in clinical practice, various factors among patients with PSCI receiving acupuncture treatment may influence the recovery of their cognitive function.

Based on the above research background, this study aims to explore factors associated with cognitive function recovery in ischemic stroke patients receiving acupuncture as an adjunctive component of multimodal inpatient rehabilitation. A total of 226 stroke patients who were hospitalized in the rehabilitation department of our hospital and received acupuncture treatment from January 2023 to December 2025 were enrolled. Baseline data of the patients were collected from the electronic medical record system, and multivariate analysis methods were employed to explore the associations between the aforementioned factors and cognitive recovery outcomes. These exploratory findings may help generate hypotheses regarding patient characteristics associated with a lower likelihood of meeting the ΔMoCA improvement threshold among patients with PSCI receiving acupuncture, and may inform the design of future prospective studies examining individualized rehabilitation protocols.

## 2 Methods

### 2.1 Study design and participants

This study was a single-center retrospective cohort study. The study participants were derived from stroke patients who were hospitalized in the rehabilitation department of our hospital and received acupuncture treatment between January 2023 and December 2025. Baseline cognitive assessment was conducted within 48 h of admission, and post-treatment assessment was performed within 48 h before discharge. All patients received standardized basic rehabilitation therapy throughout their hospitalization, with the intensity and duration determined by the rehabilitation team based on clinical need. The exact dates of stroke onset, admission, baseline assessment, acupuncture initiation, final assessment, and discharge were extracted from electronic medical records. Patient medical records meeting the inclusion and exclusion criteria were retrieved through the hospital’s electronic medical record system. The detailed study flowchart is shown in Figure 1. Of note, 12 patients who interrupted acupuncture treatment during hospitalization were excluded because the post-treatment MoCA assessment could not be completed; this may introduce attrition/selection bias, as these patients may differ systematically from those who completed the full treatment course.

**Figure 1:**
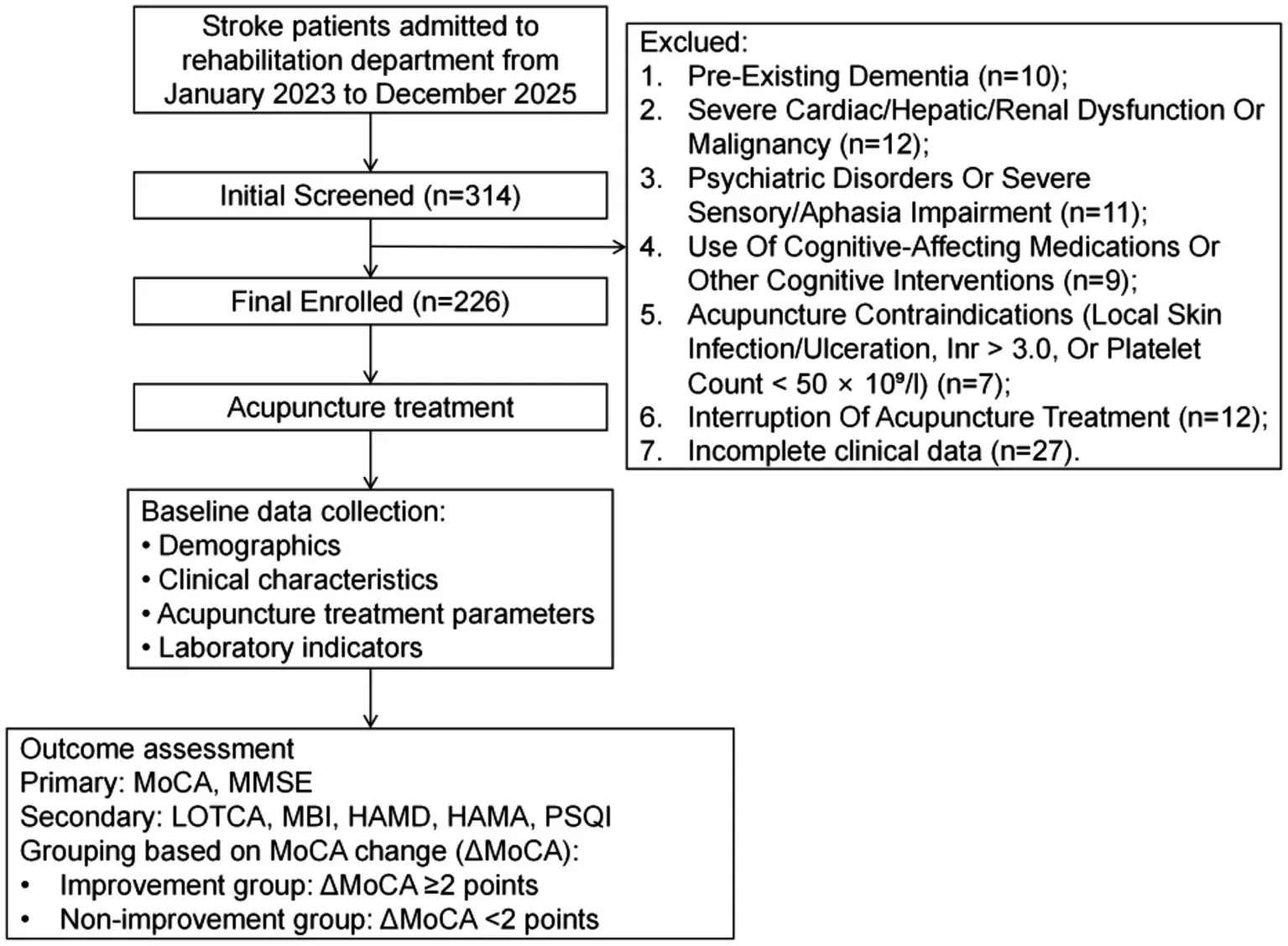
Study flowchart.

### 2.2 Inclusion criteria

(1) Age ≥ 18 years; (2) Meeting the diagnostic criteria for ischemic stroke; (3) Clinical stage classified as acute or recovery phase, i.e., onset within 6 months; (4) Presence of PSCI, defined as an admission MoCA score <26 points; (5) Receipt of regular acupuncture treatment during hospitalization.

### 2.3 Exclusion criteria

(1) Pre-existing diagnosis of dementia, Alzheimer’s disease, or cognitive dysfunction due to other causes prior to stroke; (2) Concurrent severe cardiac, hepatic, or renal dysfunction, or malignancy; (3) History of psychiatric disorders or severe visual/auditory impairment, or aphasia precluding cooperation with cognitive function assessment; (4) Concurrent use of medications known to affect cognitive function or receipt of other interventions targeting cognitive impairment during acupuncture treatment; (5) Presence of conditions that represent contraindications or precautions for acupuncture therapy, including: local skin infection or ulceration at the selected acupoints; severe bleeding disorders; ongoing anticoagulant therapy with international normalized ratio (INR) > 3.0, which is associated with a significantly increased risk of bleeding complications during needling (16); or platelet count < 50 × 10^9^/L, a threshold below which the risk of bleeding or hematoma formation is substantially elevated and acupuncture is generally not recommended (17); (6) Interruption of acupuncture treatment during hospitalization due to disease progression, transfer to another department, discharge against medical advice, or other reasons; (7) Incomplete clinical data, including missing baseline assessment, acupuncture treatment records, or follow-up cognitive function evaluation data.

### 2.4 Ethics statement

This study followed the principles of the Declaration of Helsinki and was approved by the Medical Ethics Committee of our hospital. Given that this study was a retrospective analysis of medical records and patient identifying information (including name, hospital admission number, and ID card number) was de-identified during data collection, the Ethics Committee granted a waiver of informed consent.

### 2.5 Sample size calculation

This study was a retrospective study. The sample size estimation was based on the ratio of improvement to non-improvement groups reported in previous similar studies (18). With a significance level (α) set at 0.05 and a statistical power (1-β) of 0.80, the minimum required sample size was calculated using the formula for Logistic regression analysis. Since the primary outcome event for logistic regression was non-improvement (ΔMoCA < 2 points), the event count was 78. Considering an estimated 10 candidate variables in the final model, an events-per-variable (EPV) ratio of approximately 7.8:1 was achieved. While this is slightly below the conventional 10:1 threshold, it is within acceptable limits for exploratory analyses given the prespecified exploratory nature of the study and the limited number of candidate variables. This sample size was therefore considered adequate for the present hypothesis-generating analysis, and the results should be interpreted with caution accordingly.

### 2.6 Treatment protocol

Basic Pharmacological Treatment: Standardized pharmacological management was provided for underlying diseases including hypertension, hyperlipidemia, and diabetes mellitus. According to the neurological diagnosis and treatment guidelines, conventional therapies such as antiplatelet aggregation, plaque stabilization, and improvement of cerebral blood circulation were administered. However, other medications or therapeutic interventions specifically targeting cognitive impairment were not permitted.

Basic Rehabilitation Therapy: Based on the assessment results, our rehabilitation therapists developed and implemented individualized rehabilitation programs for patients, including cognitive training, occupational therapy, exercise therapy, and physical therapy.

Acupuncture Treatment Protocol: The “Tiaoren Tongdu” (Regulating the Conception Vessel and Unblocking the Governor Vessel) acupuncture method was selected in our department. The primary acupoints included GV20 (Baihui), GV24 (Shenting), EX-HN3 (Yintang), GV26 (Renzhong), CV12 (Zhongwan), CV4 (Guanyuan), CV6 (Qihai), and GV4 (Mingmen). The adjunctive acupoints included Temporal Triple Needles (Tiaosan Zhen), as well as bilateral LI11 (Quchi), TE5 (Waiguan), LI4 (Hegu), ST36 (Zusanli), SP6 (Sanyinjiao), and LR3 (Taichong). Adjunctive acupoints were selected based on the patient’s traditional Chinese medicine syndrome differentiation and clinical manifestations, with the following syndrome patterns and their key clinical features: Spleen-Kidney deficiency: fatigue, poor appetite, lumbar soreness, cold intolerance, and frequent urination - for which BL23 (Shenshu), BL20 (Pishu), KI3 (Taixi), and KI6 (Zhaohai) were added; Liver-Kidney deficiency: dizziness, tinnitus, dry eyes, irritability, and insomnia - for which BL18 (Ganshu), BL23 (Shenshu), and GB20 (Fengchi) were added; Qi deficiency with blood stasis: fatigue, pale complexion, spontaneous sweating, and fixed stabbing pain - for which BL17 (Geshu) and SP10 (Xuehai) were added, with moxibustion applied to CV6 (Qihai) and CV4 (Guanyuan); Phlegm-stasis obstructing the collaterals: heavy sensation, dizziness, thick tongue coating, and focal neurological deficits - for which SP9 (Yinlingquan), ST40 (Fenglong), and SP10 (Xuehai) were added; Wind-phlegm obstructing the collaterals: sudden onset, vertigo, slurred speech, and thick greasy tongue coating - for which GB20 (Fengchi) and ST40 (Fenglong) were added; Phlegm-heat in the fu organs: constipation, abdominal distension, yellow thick tongue coating, and restlessness - for which ST40 (Fenglong), GB34 (Yanglingquan), SP15 (Daheng), and ST25 (Tianshu) were added.

Disposable Global brand filiform needles (specifications: 25 × 0.30 mm or 40 × 0.30 mm, Suzhou Global Acupuncture Medical Devices Co., Ltd.) were used. Specific procedures were as follows: ① Patients were placed in a supine or lateral position according to their actual condition. ② Acupuncture precautions: Strict skin disinfection was performed to prevent infection; treatment was not administered when patients were excessively fatigued, hungry, or had other contraindications to acupuncture. ③ Specific needling techniques: GV20 (Baihui) and GV24 (Shenting) were inserted subcutaneously against the direction of the Governor Vessel to a depth of 0.5–0.8 cun (approx. 10–16 mm); EX-HN3 (Yintang) was inserted subcutaneously with pinched skin to a depth of 0.3–0.5 cun (approx. 6–10 mm); CV12 (Zhongwan), CV4 (Guanyuan), and CV6 (Qihai) were inserted perpendicularly to a depth of 1–1.5 cun (approx. 20–30 mm). After achieving deqi (needling sensation), GV4 (Mingmen) and GV3 (Yaoyangguan) were inserted obliquely upward to a depth of 0.5–1 cun (approx. 10–20 mm). LI11 (Quchi), TE5 (Waiguan), LI4 (Hegu), and LR3 (Taichong) were inserted perpendicularly to a depth of 0.5–0.8 cun (approx. 10–16 mm); ST36 (Zusanli) and SP6 (Sanyinjiao) were inserted perpendicularly to a depth of 1–1.5 cun (approx. 20–30 mm). The remaining acupoints were needled according to routine acupuncture procedures. ④ Treatment duration: Once daily during hospitalization, with each session lasting 30 min.

Treatment schedule and dose calculation: All patients were prescribed a standardized once-daily acupuncture protocol during hospitalization. Actual weekly frequency varied due to non-clinical factors (national holidays, patient-requested breaks, and minor adverse reactions), and was calculated as total completed sessions divided by weeks of hospitalization, with missed sessions counted as zero-dose days. Total treatment course was defined as the interval from first to last session, irrespective of missed sessions. Deviations from the planned schedule were driven primarily by non-clinical factors. Adjunctive acupoint selection varied by syndrome differentiation, introducing additional heterogeneity not captured by frequency or duration; these clinically individualized selections were not treated as independent exposure variables but are acknowledged as potential contributors to between-group differences.

### 2.7 Clinical outcome measures

#### 2.7.1 Primary outcome measures

1. Montreal Cognitive Assessment (MoCA) (19): Rapid screening for mild cognitive impairment. Total score 30, covering eight domains: visuospatial/executive function, naming, memory, attention, language, abstract thinking, delayed recall, and orientation. Add 1 point if education ≤12 years; ≥26 indicates normal cognition. The Beijing version of the MoCA was used in this study, and the same version was administered at both baseline and follow-up assessments.
2. Mini-Mental State Examination (MMSE) (20): Assesses global cognition. Total score 30, comprising orientation (10 points), registration (3), attention/calculation (5), recall (3), and language (9). Cognitive dysfunction indicated by scores ≤17 (illiterate), ≤20 (primary school), or ≤24 (middle school+).

#### 2.7.2 Secondary outcome measures

1. Loewenstein Occupational Therapy Cognitive Assessment (LOTCA) (21): Cognitive-perceptual function across 7 domains (Orientation, Visual Perception, Spatial Perception, Motor Praxis, Visuomotor Organization, Thinking Operations, Attention & Concentration). Total: 26–115 (higher = better).
2. Modified Barthel Index (MBI) (22): ADL across 10 items (feeding, bathing, grooming, dressing, bowel/bladder control, toilet use, bed-chair transfer, ambulation, stairs). Total: 100.
3. Hamilton Depression Rating Scale (HAMD) (23): Depression severity. 0–7 none, 8–16 mild, 17–23 moderate, ≥24 severe.
4. Hamilton Anxiety Rating Scale (HAMA) (24): Anxiety severity. 0–7 none, 8–14 mild, 15–21 moderate, 22–29 marked, ≥30 severe.
5. Pittsburgh Sleep Quality Index (PSQI) (25): One-month sleep quality across 7 components. Score >5 (max 21) = poor sleep.

#### 2.7.3 Grouping criteria

For the purpose of descriptive subgroup comparison and exploratory logistic regression analysis, patients were operationally classified into an “improvement group” (ΔMoCA ≥ 2 points) and a “non-improvement group” (ΔMoCA < 2 points) based on the change in MoCA score. The 2-point threshold was chosen with reference to previous literature on minimal clinically important differences (26, 27); however, it is important to note that this cut-off is used as a statistical stratification variable for this retrospective study rather than as a confirmed clinical definition of cognitive recovery. Given the potential influence of measurement error, repeated-testing effects, and regression to the mean, this grouping should be interpreted as an operational and exploratory definition of improvement, and the group comparisons presented below are descriptive in nature.

### 2.8 Data collection

1. General demographic characteristics: age, sex, body mass index (BMI), marital status, years of education, family history of stroke, smoking history, alcohol consumption history;
2. Clinical characteristics: infarct location, hypertension, diabetes mellitus, hyperlipidemia, hyperuricemia, homocysteine, hemoglobin;
3. Acupuncture treatment parameters: time to acupuncture intervention, treatment frequency (sessions per week), total treatment course (weeks).
4. Temporal variables: length of hospital stay (weeks) and total duration of concurrent rehabilitation therapies (cognitive training, occupational therapy, exercise therapy, and physical therapy; hours).

For laboratory variables, all were extracted from electronic medical records as part of routine admission testing and analyzed as categorical variables based on predefined diagnostic criteria as follows: hyperlipidemia was defined as total cholesterol ≥ 5.72 mmol/L and/or triglycerides ≥ 1.70 mmol/L, or documented diagnosis; hyperuricemia was defined as serum uric acid > 420 μmol/L; elevated homocysteine was defined as > 15 μmol/L; and low hemoglobin was defined as < 130 g/L in males or < 120 g/L in females.

### 2.9 Statistical analysis

Statistical analysis used SPSS 26.0. Normally distributed continuous data were reported as mean ± SD and compared using the independent t-test; non-normally distributed data as median (IQR) and compared using the Mann–Whitney U test. Categorical data were reported as n (%) and compared using chi-square or Fisher’s exact test. For longitudinal comparisons of cognitive and functional outcomes (MoCA, MMSE, LOTCA, MBI, HAMD, HAMA, and PSQI), we employed a two-step analytical approach. First, within-group pre−/post-treatment changes were assessed using paired *t*-tests for normally distributed continuous variables and Wilcoxon signed-rank tests for non-normally distributed variables. Second, and critically, between-group differences in the magnitude of change (ΔScore = Post-treatment - Pre-treatment) were directly compared using independent t-tests or Mann–Whitney *U* tests, as appropriate. This approach directly tests whether the degree of improvement differed between the improvement and non-improvement groups, rather than relying on the informal comparison of separate within-group P.

Improvement in MoCA score was the dependent variable, coded as “non-improvement” (ΔMoCA<2 points). We adopted a clinically informed, exploratory multivariable logistic regression model. Clinically relevant variables were selected based on clinical relevance and prior literature, rather than solely on univariate significance, and were entered simultaneously into the model. Two temporal covariates—time from stroke onset to baseline assessment and interval between baseline and final assessment—were forced into the model to adjust for potential confounding. The covariate set should therefore be regarded as clinically informed rather than prespecified. Univariate analyses were performed for descriptive purposes only and were not used as a screening step. Results were expressed as odds ratios (OR) with 95% confidence intervals (CI); an OR > 1 indicates higher odds of non-improvement, while an OR<1 indicates lower odds of non-improvement. The linearity-in-the-logit assumption for continuous predictors was assessed using the Box–Tidwell approach, in which an interaction term between each continuous predictor and its natural logarithm was added to the model; a non-significant interaction term was taken to indicate no evidence of violation. Because the model was constructed for explanatory (associational) rather than predictive purposes, discrimination metrics (e.g., apparent c-statistic/AUC) were not calculated, and model performance claims were correspondingly limited. Model calibration was examined descriptively using the Hosmer-Lemeshow test, with the caveat that a non-significant result does not by itself demonstrate good calibration. All tests were two-tailed with α = 0.05. Given the exploratory nature of this study and the multiplicity of secondary outcome comparisons, we did not adjust for multiple comparisons; instead, all secondary outcome analyses are explicitly designated as exploratory, and results should be interpreted with caution. No adjustment for multiplicity was applied for secondary outcomes to avoid inflating Type II error in this hypothesis-generating analysis.

## 3 Results

### 3.1 General demographic characteristics

A total of 226 PSCI patients receiving acupuncture treatment were enrolled. Based on the improvement in MoCA score after treatment, patients were divided into improvement group (ΔMoCA ≥ 2 points, *n* = 148, 65.49%) and non-improvement group (ΔMoCA < 2 points, *n* = 78, 34.51%). Table 1 compares the general demographic characteristics between two groups. The results showed that the mean age of patients in the improvement group was significantly lower than that in the non-improvement group (58.22 years vs. 63.12 years), and the median years of education was higher (10 years vs. 7 years), with both differences reaching statistical significance (*p* < 0.001). There were no significant differences in terms of sex, marital status, body mass index, family history of stroke, smoking history, or alcohol consumption history, suggesting that age and education level were demographic factors associated with the likelihood of being in the ΔMoCA ≥2 subgroup in this cohort.

**Table 1:** Comparison of general demographic characteristics.

Variable	Total (*n* = 226)	Improvement group (*n* = 148)	Non-improvement group (*n* = 78)	Statistic	*P*
Sex, *n* (%)				χ^2^ = 0.527	0.468
Male	132 (58.41)	89 (60.14)	43 (55.13)		
Female	94 (41.59)	59 (39.86)	35 (44.87)		
Age (years), mean ± SD	59.91 ± 6.46	58.22 ± 6.42	63.12 ± 5.22	*t* = −5.802	<0.001
BMI (kg/m^2^), mean ± SD	24.20 ± 2.44	24.12 ± 2.33	24.37 ± 2.65	*t* = −0.728	0.468
Marital status, *n* (%)				χ^2^ = 0.693	0.405
Married	178 (78.76)	119 (80.41)	59 (75.64)		
Unmarried/Divorced/Widowed	48 (21.24)	29 (19.59)	19 (24.36)		
Years of education, median (IQR)	9.00 (7.00, 11.00)	10.00 (8.00, 12.00)	7.00 (5.00, 9.75)	Z = 6.269	<0.001
Family history of stroke, *n* (%)				χ^2^ = 1.761	0.184
Yes	26 (11.50)	14 (9.46)	12 (15.38)		
No	200 (88.50)	134 (90.54)	66 (84.62)		
Smoking history, *n* (%)				χ^2^ = 0.446	0.504
Yes	86 (38.05)	54 (36.49)	32 (41.03)		
No	140 (61.95)	94 (63.51)	46 (58.97)		
Alcohol consumption history, *n* (%)				χ^2^ = 0.441	0.507
Yes	69 (30.53)	43 (29.05)	26 (33.33)		
No	157 (69.47)	105 (70.95)	52 (66.67)		

Data are presented as mean ± SD, median (IQR), or n (%), as appropriate.

### 3.2 Clinical characteristics

Table 2 compares the clinical characteristics of patients. The median admission NIHSS score in the improvement group was significantly lower than that in the non-improvement group (6 points vs. 7 points, *p* < 0.001), and the proportion of patients with comorbid diabetes mellitus was markedly lower (27.03% vs. 53.85%, *p* < 0.001). There were no significant differences in terms of infarct location distribution, hypertension, hyperlipidemia, hyperuricemia, homocysteine, or hemoglobin levels, suggesting that the severity of neurological deficit and diabetes status may be closely associated with cognitive recovery.

**Table 2:** Comparison of clinical characteristics.

Variable	Total (*n* = 226)	Improvement group (*n* = 148)	Non-improvement group (*n* = 78)	Statistic	*p-*value
Infarct location, *n* (%)				χ^2^ = 0.789	0.430
Basal ganglia	80 (35.40)	55 (37.16)	25 (32.05)		
Corona radiata	5 (2.21)	3 (2.03)	2 (2.56)		
Periventricular	7 (3.10)	4 (2.70)	3 (3.85)		
Brainstem	23 (10.18)	15 (10.14)	8 (10.26)		
Cerebral lobe	28 (12.39)	20 (13.51)	8 (10.26)		
Thalamus	12 (5.31)	7 (4.73)	5 (6.41)		
Cerebellum	4 (1.77)	3 (2.03)	1 (1.28)		
Centrum semiovale	3 (1.33)	1 (0.68)	2 (2.56)		
Multiple infarcts	64 (28.32)	40 (27.03)	24 (30.77)		
Admission NIHSS score, median (IQR)	6.00 (5.00, 9.00)	6.00 (4.00, 8.00)	7.00 (6.00, 9.00)	Z = -4.127	<0.001
Hypertension, *n* (%)				χ^2^ = 0.201	0.654
Yes	158 (69.91)	102 (68.92)	56 (71.79)		
No	68 (30.09)	46 (31.08)	22 (28.21)		
Diabetes mellitus, *n* (%)				χ^2^ = 15.892	<0.001
Yes	82 (36.28)	40 (27.03)	42 (53.85)		
No	144 (63.72)	108 (72.97)	36 (46.15)		
Hyperlipidemia, *n* (%)				χ^2^ = 0.265	0.607
Yes	98 (43.36)	66 (44.59)	32 (41.03)		
No	128 (56.64)	82 (55.41)	46 (58.97)		
Hyperuricemia, *n* (%)				χ^2^ = 0.000	0.986
Yes	52 (23.01)	34 (22.97)	18 (23.08)		
No	174 (76.99)	114 (77.03)	60 (76.92)		
Homocysteine, *n* (%)				χ^2^ = 0.567	0.451
Normal	111 (49.12)	70 (47.30)	41 (52.56)		
Elevated	115 (50.88)	78 (52.70)	37 (47.44)		
Hemoglobin, *n* (%)				χ^2^ = 1.825	0.177
Normal	137 (60.62)	85 (57.43)	52 (66.67)		
Low	89 (39.38)	63 (42.57)	26 (33.33)		

Data are presented as median (IQR), or n (%), as appropriate.

### 3.3 Acupuncture treatment parameters

Table 3 analyzes the influence of acupuncture treatment-related parameters on cognitive recovery. The median time from stroke onset to acupuncture intervention was significantly shorter in the improvement group compared with the non-improvement group (*p* < 0.001). The median treatment frequency was higher in the improvement group (6 sessions/week vs. 4 sessions/week, *p* < 0.001), and the mean total treatment course was also longer (6.21 weeks vs. 4.22 weeks, *p* < 0.001).

**Table 3:** Comparison of acupuncture treatment parameters.

Variable	Total (*n* = 226)	Improvement group (*n* = 148)	Non-improvement group (*n* = 78)	Statistic	*P-*value
Time from stroke onset to acupuncture intervention (days)	12.00 (8.00, 17.00)	11.00 (6.00, 15.00)	17.00 (11.00, 18.00)	*Z* = –4.715	<0.001
Treatment frequency (sessions per week)	5.00 (4.00, 6.00)	6.00 (5.00, 7.00)	4.00 (4.00, 5.00)	*Z* = –7.514	<0.001
Total treatment course (weeks)	5.52 ± 1.58	6.21 ± 1.22	4.22 ± 1.35	*t* = 11.199	<0.001

Data are presented as mean ± SD, median (IQR), or n (%), as appropriate.

### 3.4 Analysis of temporal and concurrent rehabilitation variables

As shown in Table 4, there was no significant difference in length of hospital stay, total rehabilitation hours, time from stroke onset to baseline assessment, or interval between baseline and final assessment between the two groups (all *p* > 0.05). However, these between-group comparisons do not rule out residual confounding. First, total rehabilitation hours were aggregated across cognitive training, occupational therapy, exercise therapy, and physical therapy, and our electronic medical records did not capture the duration of each component separately. Cognitive-specific training is particularly relevant to MoCA outcomes, and its potential imbalance between groups cannot be excluded. Second, these comparisons are descriptive and do not adjust for unmeasured factors such as patient motivation, family support, or early clinical response, which may have influenced both treatment intensity and cognitive outcomes.

**Table 4:** Comparison of temporal and concurrent rehabilitation variables.

Variable	Total (*n* = 226)	Improvement group (*n* = 148)	Non-improvement group (*n* = 78)	Statistic	*P-*value
Length of hospital stay (weeks)	7.00 (6.00, 8.00)	7.00 (6.00, 8.00)	7.00 (6.00, 8.00)	*Z* = 1.177	0.239
Rehabilitation hours	41.54 ± 9.24	42.18 ± 9.55	40.33 ± 8.54	*t* = 1.434	0.153
Time from stroke onset to baseline assessment (days)	16.00 (13.00, 18.00)	15.00 (12.00, 18.00)	16.00 (13.00, 18.00)	*Z* = –0.848	0.396
Interval between baseline and final assessment (days)	51.50 (44.00, 63.00)	54.00 (44.00, 65.00)	48.50 (43.00, 57.50)	*Z* = –1.862	0.063

Data are presented as mean ± SD, median (IQR), or n (%), as appropriate.

### 3.5 Changes in cognitive function and related scale scores

#### 3.5.1 Primary outcome measures

Table 5 shows the changes in MoCA and MMSE scores before and after treatment. In the improvement group, both MoCA and MMSE scores after treatment were significantly higher than those before treatment (*p* < 0.001), whereas no significant improvement was observed in the non-improvement group. Intergroup comparison showed that after treatment, both MoCA and MMSE scores in the improvement group were significantly higher than those in the non-improvement group (*p* < 0.001).

**Table 5:** Comparison of primary outcome measures.

Scale	Time	Improvement group (*n* = 148)	Non-improvement group (*n* = 78)	Between-group *t-*value	Between-group *P-*value	Mean difference (95% CI)
MoCA	Pre-treatment	19.03 ± 2.46	19.24 ± 2.88	*t* = −0.574	0.567	−0.210 (−0.931 to 0.511)
	Post-treatment	21.44 ± 2.68	19.85 ± 2.99	*t* = 4.077	<0.001	1.593 (0.823 to 2.363)
	Within-group *t-*value	8.040	1.280			
	Within-group *P-*value	<0.001	0.202			
	Mean difference (95% CI)	2.405 (1.817 to 2.994)	0.603 (−0.327 to 1.532)			
MMSE	Pre-treatment	20.42 ± 2.58	20.33 ± 2.92	*t* = 0.226	0.821	0.086 (−0.659 to 0.831)
	Post-treatment	23.18 ± 2.59	20.74 ± 2.93	*t* = 6.428	<0.001	2.439 (1.691 to 3.186)
	Within-group *t-*value	9.197	0.876			
	Within-group *P-*value	<0.001	0.383			
	Mean difference (95% CI)	2.764 (2.172 to 3.355)	0.410 (−0.515 to 1.336)			

Data are presented as mean ± SD.

Critically, direct comparison of the magnitude of change (ΔScore) between groups demonstrated that the improvement group exhibited significantly greater improvements than the non-improvement group for both MoCA and MMSE (*p* < 0.001) (see Table 6).

**Table 6:** Comparison of pre-to-post treatment change scores for MoCA and MMSE.

Indicator	Improvement group (*n* = 148)	Non-improvement group (*n* = 78)	*Z*-value	*p*-value
ΔMoCA	2.00 (2.00, 2.00)	1.00 (0.00, 1.00)	*Z* = 13.447	<0.001
ΔMMSE	2.00 (2.00, 4.00)	1.00 (0.00, 1.00)	*Z* = 12.701	<0.001

Δ = post-treatment value minus pre-treatment value.

#### 3.5.2 Secondary outcome measures

Table 7 summarizes the changes in secondary outcome measures. The improvement group showed significant improvements in LOTCA, MBI, HAMD, HAMA, and PSQI scores after treatment compared with before treatment (*p* < 0.05), whereas the non-improvement group showed only slight improvements in MBI, HAMD, HAMA, and PSQI, with no significant change in LOTCA.

**Table 7:** Comparison of secondary outcome measures.

Scale	Time	Improvement group (*n* = 148)	Non-improvement group (*n* = 78)	Between-group *t-*value	Between-group *p-*value	Mean difference (95% CI)
LOTCA	Pre-treatment	63.20 ± 12.58	61.03 ± 14.20	*t* = 1.179	0.240	2.170 (−1.458 to 5.798)
	Post-treatment	74.32 ± 12.59	64.01 ± 15.38	*t* = 5.410	<0.001	10.305 (6.551 to 14.058)
	Within-group *t-*value	7.601	1.261			
	Within-group *P-*value	<0.001	0.209			
	Mean difference (95% CI)	11.122 (8.242 to 14.001)	2.987 (−1.694 to 7.668)			
MBI	Pre-treatment	58.21 ± 14.46	58.46 ± 15.53	*t* = −0.121	0.903	−0.252 (−4.343 to 3.839)
	Post-treatment	68.48 ± 14.81	65.90 ± 16.03	*t* = 1.211	0.227	2.582 (−1.620 to 6.784)
	Within-group *t-*value	6.036	2.943			
	Within-group *P-*value	<0.001	0.004			
	Mean difference (95% CI)	10.270 (6.922 to 13.619)	7.436 (2.444 to 12.428)			
HAMD	Pre-treatment	12.48 ± 3.05	12.38 ± 3.43	t = 0.213	0.831	0.095 (−0.784 to 0.974)
	Post-treatment	9.11 ± 3.27	10.59 ± 4.31	t = −2.879	0.004	−1.475 (−2.484 to −0.466)
	Within-group *t-*value	9.160	2.704			
	Within-group *P-*value	<0.001	0.008			
	Mean Difference (95% CI)	−3.365 (−4.088 to −2.642)	−1.795 (−3.028 to −0.561)			
HAMA	Pre-treatment	11.80 ± 3.01	11.50 ± 2.57	t = 0.741	0.460	0.297 (−0.494 to 1.088)
	Post-treatment	8.47 ± 3.17	10.26 ± 3.73	t = −3.782	<0.001	−1.783 (−2.713 to −0.854)
	Within-group *t-*value	9.252	2.426			
	Within-group *P-*value	<0.001	0.017			
	Mean difference (95% CI)	−3.324 (−4.031 to −2.617)	−1.244 (−2.256 to −0.231)			
PSQI	Pre-treatment	12.60 ± 2.73	12.13 ± 3.14	t = 1.175	0.241	0.473 (−0.321 to 1.267)
	Post-treatment	8.77 ± 3.08	9.91 ± 3.21	t = −2.608	0.010	−1.140 (−2.001 to −0.279)
	Within-group *t-*value	11.320	4.364			
	Within-group *P-*value	<0.001	<0.001			
	Mean difference (95% CI)	−3.831 (−4.497 to −3.165)	−2.218 (−3.222 to −1.214)			

Data are presented as mean ± SD.

Direct between-group comparison of the magnitude of change (ΔScore) revealed that the improvement group showed significantly greater improvements than the non-improvement group for LOTCA, HAMA, MBI, HAMD, and PSQI (*p* < 0.001) (see Table 8).

**Table 8:** Comparison of pre-to-post treatment change scores for LOTCA, MBI, HAMD, HAMA and PSQI.

Indicator	Improvement group (*n* = 148)	Non-improvement group (*n* = 78)	*Z*-value	*p*-value
ΔLOTCA	10.00 (7.00, 15.00)	5.00 (3.00, 5.00)	*Z* = 12.635	<0.001
ΔMBI	10.00 (10.00, 10.00)	10.00 (5.00, 10.00)	*Z* = 5.723	<0.001
ΔHAMD	−4.00 (−4.00, −2.00)	−4.00 (−4.00, 1.75)	*Z* = –3.271	0.001
ΔHAMA	−4.00 (−4.00, −2.00)	−2.00 (−4.00, 3.00)	*Z* = -5.612	<0.001
ΔPSQI	−3.50 (−7.00, −1.00)	−2.00 (−5.00, 0.75)	*Z* = -2.176	0.030

Δ = post-treatment value minus pre-treatment value.

### 3.6 Logistic regression analysis of factors influencing cognitive recovery

Using non-improvement (ΔMoCA < 2) as the dependent event, we first performed univariate logistic regression analyses to examine the crude associations of each candidate variable with the outcome (Table 9). Subsequently, a multivariable logistic regression model was constructed including clinically relevant variables selected based on clinical relevance and prior literature, together with two temporal covariates (time from stroke onset to baseline assessment and interval between baseline and final assessment) that were forced into the model to adjust for potential confounding (Table 10). In the univariate analyses, age, education years, admission NIHSS score, diabetes mellitus, time to acupuncture intervention, treatment frequency, and total treatment course were significantly associated with the outcome (*p* < 0.05).

**Table 9:** Univariate logistic regression analysis of factors associated with non-improvement in MoCA score.

Variable	*β*	S.E	*Z*	*P*	OR (95%CI)
Age (years)	0.144	0.028	5.112	<0.001	1.155 (1.093–1.221)
Years of education	−0.342	0.059	−5.781	<0.001	0.710 (0.632–0.798)
Admission NIHSS score	0.268	0.065	4.142	<0.001	1.308 (1.152–1.485)
Diabetes mellitus	1.147	0.293	3.916	<0.001	3.150 (1.774–5.594)
Acupuncture intervention time (days)	0.128	0.028	4.519	<0.001	1.137 (1.075–1.202)
Treatment frequency (sessions/week)	−1.015	0.153	−6.653	<0.001	0.362 (0.269–0.489)
Total treatment course (weeks)	−1.285	0.183	−7.015	<0.001	0.277 (0.193–0.396)
Time from stroke onset to baseline assessment (days)	0.017	0.028	0.600	0.548	1.017 (0.963–1.074)
Interval between baseline and final assessment (days)	−0.016	0.011	−1.488	0.137	0.984 (0.964–1.005)
Baseline MoCA	0.031	0.054	0.575	0.565	1.031 (0.928–1.146)

**Table 10:** Multivariable logistic regression analysis of factors associated with non-improvement in MoCA score.

Variable	*β*	S.E	*Z*	*P*	OR (95%CI)	VIF
Age (years)	0.189	0.057	3.294	<0.001	1.208 (1.079–1.352)	1.111
Years of education	−0.301	0.111	−2.723	0.006	0.740 (0.596–0.919)	1.207
Admission NIHSS score	0.497	0.156	3.188	0.001	1.643 (1.211–2.230)	1.048
Diabetes mellitus	1.555	0.659	2.360	0.018	4.737 (1.302–17.236)	1.139
Acupuncture intervention time (days)	0.150	0.059	2.556	0.011	1.162 (1.036–1.303)	1.148
Treatment frequency (sessions/week)	−1.304	0.291	−4.480	<0.001	0.271 (0.153–0.480)	1.145
Total treatment course (weeks)	−1.394	0.286	−4.872	<0.001	0.248 (0.142–0.435)	1.271
Time from stroke onset to baseline assessment (days)	0.030	0.057	0.517	0.605	1.030 (0.920–1.153)	1.032
Interval between baseline and final assessment (days)	−0.037	0.023	−1.593	0.111	0.964 (0.921–1.008)	1.020
Baseline MoCA	0.102	0.116	0.882	0.378	1.107 (0.883–1.389)	1.039

Multivariate analysis demonstrated that advanced age (OR = 1.208, *p* < 0.001), higher NIHSS score (OR = 1.643, *p* = 0.001), longer time from stroke onset to acupuncture intervention (OR = 1.162, *p* = 0.011) and diabetes mellitus (OR = 4.737, *p* = 0.018) were risk factors for poor cognitive recovery. In contrast, longer education duration (OR = 0.740, *p* = 0.006), higher treatment frequency (OR = 0.271, *p* < 0.001), and longer treatment course (OR = 0.248, *p* < 0.001) were associated with a higher probability of meeting the ΔMoCA ≥ 2 improvement threshold in this exploratory analysis. Notably, time from stroke onset to baseline assessment (OR = 1.030, *p* = 0.605), the interval between baseline and final assessment (OR = 0.964, *p* = 0.111) and the baseline MoCA (OR = 1.107, *p* = 0.378) were not associated with cognitive recovery after adjustment for other covariates. The linearity-in-the-logit assumption was examined using the Box–Tidwell approach; none of the interaction terms between continuous predictors and their natural logarithms was statistically significant (all *p* > 0.05), providing no evidence of violation of this assumption. The Hosmer–Lemeshow test was not statistically significant (χ^2^ = 10.079, *p* = 0.260), indicating no evidence of gross miscalibration; however, this test has limited power, and a non-significant result should not be interpreted as demonstrating good calibration. Because the model was intended for explanatory (associational) rather than predictive purposes, discrimination metrics such as the apparent c-statistic/AUC were not calculated, and no claims regarding predictive performance are made.

### 3.7 Safety and adverse events

Based on systematic review of electronic medical records and daily nursing documentation, no acupuncture-related serious adverse events—including pneumothorax, vasovagal syncope, significant hemorrhage, needle breakage, or local infection—were recorded during the entire hospitalization period. Minor adverse events, such as transient local subcutaneous hematoma (petechiae or ecchymosis) or mild needling pain, were noted informally in some nursing records. However, these events were self-limiting, required no medical intervention.

## 4 Discussion

PSCI remains one of the most disabling sequelae of cerebrovascular events, substantially delaying functional recovery and compromising long-term quality of life (28). Acupuncture has gained increasing recognition as an effective adjunctive therapy in stroke rehabilitation, yet the heterogeneity in patient responses underscores the urgent need to identify modifiable and non-modifiable determinants of treatment outcome (29). In this retrospective cohort of 226 PSCI patients receiving acupuncture as part of an inpatient rehabilitation program, we found that earlier initiation, higher weekly session frequency, and longer total treatment duration were exploratorily associated with favorable cognitive recovery. These exploratory findings may help generate hypotheses for optimizing treatment strategies and may assist in the prospective identification of patients at high risk for poor prognosis, although these associations require validation in future controlled studies. Consistent with recent international meta-analyses, a systematic review of 29 RCTs (2,477 patients) found significantly higher MMSE and MoCA scores with acupuncture, supporting its effectiveness for post-stroke cognitive impairment (15). Another meta-analysis of 24 studies involving 1,769 patients similarly confirmed that electroacupuncture significantly improves cognitive function in patients with PSCI, with pooled effect sizes of 2.62 for MMSE and 3.01 for MoCA (30). Building on this evidence, the present exploratory study further identified factors associated with cognitive improvement in patients receiving acupuncture within a multimodal rehabilitation setting, thereby helping to generate hypotheses for future studies on individualized acupuncture for PSCI.

Age was associated with lower odds of meeting the ΔMoCA threshold. This association may be explained by age-related declines in neuroplasticity, reduced cerebrovascular reserve capacity, and altered neuroinflammatory responses. A study by Zhou et al. (31) reported that age ≥60 years is a significant risk factor for PSCI (OR = 6.806; 95% CI: 4.203–11.023), suggesting that older patients exhibit significantly lower tolerance to ischemic injury due to decreased cerebral reserve, diminished neuroplasticity, and impaired cerebrovascular autoregulation. From a neurobiological perspective, aging is accompanied by a reduction in neuron numbers, decreased synaptic density, disruption of white matter integrity, and reduced expression of brain-derived neurotrophic factor. These pathophysiological changes collectively contribute to a poorer baseline condition for cognitive recovery in elderly stroke patients (32, 33). Furthermore, older patients often present with multiple chronic conditions, such as hypertension, diabetes mellitus, and atherosclerosis, which further exacerbate the burden of cerebrovascular pathology and create a vicious cycle (34–36). A network meta-analysis by Niu et al. (37), which included 23 randomized controlled trials, also indicated that age is an important effect modifier influencing the efficacy of non-pharmacological interventions, with older patients generally showing lower responsiveness to rehabilitation interventions compared with younger patients. These findings suggest that elderly patients with PSCI may represent a subgroup with lower baseline cognitive reserve, which could be a focus for future studies examining whether more intensive or prolonged rehabilitation protocols are beneficial in this population. Whether such strategies would improve outcomes remains to be tested in prospective trials. The mean ages were 58.22 (improvement) and 63.12 (non-improvement) years—a statistically significant difference, though both represent a relatively younger elderly population. Whether the cognitive improvement effects of acupuncture are similarly observed in older stroke patients (e.g., those over 75 years of age) remains to be validated through studies specifically targeting this population.

Years of education was significantly associated with favorable cognitive outcomes in this study. According to the cognitive reserve theory, higher educational levels can enhance the brain’s compensatory capacity against pathological damage by increasing the complexity and redundancy of neural networks, thereby preserving relatively better cognitive function despite comparable degrees of brain injury (38). Prolonged learning and intellectual engagement promote synaptogenesis, enhance neurotransmitter efficiency, and upregulate neurotrophic support, thereby providing a structural and functional buffer against ischemic disruption (39). In addition, better-educated patients typically exhibit superior health literacy and treatment adherence, which may amplify the therapeutic benefits of acupuncture through improved engagement in concurrent cognitive training and lifestyle modifications. This multidimensional advantage suggests that education should be considered not merely as a demographic covariate but as an important marker of cognitive reserve that informs prognosis and individualized rehabilitation planning.

The admission NIHSS score not only reflects the overall degree of neurological deficits but also includes cognitive-related dimensions such as level of consciousness, orientation, and language function, thereby sharing intrinsic pathophysiological links with post-stroke cognitive outcomes. A study by Kauranen et al. (40) reported that the discharge NIHSS score was strongly correlated with cognitive impairment: among patients with intact NIHSS scores, 41% exhibited cognitive deficits, whereas among patients with NIHSS scores ≥4, all exhibited cognitive deficits. A study by Cumming et al. (41) further confirmed that the NIHSS score is an independent predictor of dementia at 18 months post-stroke. A high NIHSS score often indicates a larger infarct volume, involvement of key cognitive-related brain regions such as the left frontotemporal lobe or thalamus, or the presence of multiple infarcts. These anatomical features directly disrupt the structural integrity of cognitive neural networks, leading to reduced information processing efficiency (42). From a mechanistic standpoint, acupuncture may partially restore this disrupted connectivity through somatosensory afferent signaling that engages the brainstem reticular formation, limbic system, and default-mode network. However, the extent of this restoration is inherently constrained by the baseline structural damage, highlighting the importance of early neuroimaging-based stratification.

Diabetes showed the strongest association with non-improvement, an association that warrants further investigation. The mechanisms through which diabetes exerts negative effects on cognitive function involve multiple levels of pathophysiological processes, including chronic hyperglycemia-induced accumulation of advanced glycation end products (AGEs), enhanced oxidative stress, cerebrovascular endothelial dysfunction, and cerebral microcirculatory hypoperfusion (43, 44). Chronic hyperglycemia can induce oxidative stress through the accumulation of AGEs, impairing synaptic structure and function in key cognitive brain regions such as the hippocampus (45). Diabetes-related microvascular pathology may exacerbate white matter injury and disrupt the structural integrity of cognitive neural networks (46). Furthermore, insulin resistance in diabetic patients may be associated with dysfunction of insulin signaling pathways in the brain, which play an important regulatory role in synaptic plasticity and memory formation (47). A neuroimaging systematic review by Yang et al. (48) on acupuncture for PSCI indicated that patients with diabetes exhibit abnormal patterns of brain functional network connectivity, which may reduce their responsiveness to needling interventions. Our finding that 53.85% of the non-improvement group had diabetes, versus only 27.03% in the improvement group, suggests that diabetes may be associated with a lower likelihood of cognitive improvement in this cohort. This observation, if validated in future controlled studies, could inform the design of future studies examining stratified rehabilitation protocols.

In this exploratory analysis, earlier acupuncture initiation was associated with cognitive improvement in this cohort. This observation may inform hypotheses regarding the timing of acupuncture in post-stroke cognitive rehabilitation. Recovery of cognitive function after stroke follows a specific time-window pattern, with the acute phase and early recovery phase representing the most active periods of neuroplasticity. During this period, widespread spontaneous repair mechanisms exist in the brain, including neurogenesis, synaptic remodeling, and neural network reorganization (49). Acupuncture, as a form of peripheral somatosensory stimulation, can transmit neural signals to higher limbic and cortical regions by activating relay structures in the spinal cord and brainstem, thereby modulating the functional activity of large-scale cognitive networks. A study by Li et al. (50) indicated that in the early stage following cerebral ischemia, the perilesional area exhibits dynamic changes in neurovascular unit integrity, and intervention during this time can maximize the utilization of endogenous neural repair mechanisms. After adjusting for time from stroke onset to baseline assessment and the assessment interval in the multivariate model, the association between earlier acupuncture initiation and cognitive improvement remained significant. However, this does not exclude the possibility that the association is explained by residual confounding, including unmeasured factors related to spontaneous recovery or differential follow-up duration.

Treatment frequency and total course duration, as core parameters reflecting the intensity of acupuncture therapy, both demonstrated significant associations with cognitive improvement in this study. This finding aligns with the fundamental principle that neuroplasticity depends on repetitive stimulation-induced long-term potentiation. The regulatory effect of acupuncture on synaptic plasticity is a biological process requiring cumulative effects. A meta-analysis by Zhu et al. (30) on electroacupuncture for PSCI found, in subgroup analyses stratified by treatment duration, that the 4-week subgroup showed significant heterogeneity in MoCA scores (*I*^2^ = 90.7%), whereas the 8-week subgroup exhibited extremely low heterogeneity (*I*^2^ = 0%), suggesting that treatment duration is a source of heterogeneity in MoCA scores. If confirmed in future prospective studies, these exploratory findings would suggest that treatment protocols ensuring adequate session frequency and total duration might be associated with better cognitive outcomes. However, given the observational design and the strong effect sizes observed, we caution against direct clinical translation at this stage. These results should primarily serve to inform the design of future randomized controlled trials with standardized, fixed-dose acupuncture protocols. However, whether there is a diminishing marginal effect, i.e., a plateau, with increasing treatment frequency and prolonged course duration remains an important question worthy of further investigation.

A critical caveat must be acknowledged regarding the interpretation of treatment frequency and total course duration as exposure variables. Unlike age, education, or admission NIHSS score, these parameters were not baseline characteristics but were determined during the patient’s hospitalization, making them susceptible to confounding by indication and reverse causation—patients who were clinically stable, adherent, or showing early signs of improvement may have been more likely to receive more frequent or prolonged acupuncture sessions, whereas those with complications, poor tolerance, or deteriorating status may have received abbreviated regimens. Moreover, all patients received concurrent multimodal rehabilitation, and our electronic medical records did not allow disaggregation of cognitive-specific training from other therapy components (e.g., occupational therapy, exercise therapy, or physical therapy). Although our analysis showed that length of hospital stay and total rehabilitation hours did not differ between groups, we cannot exclude the possibility that unmeasured factors—including early clinical response, patient motivation, family support, or intercurrent events—influenced both treatment intensity and cognitive outcomes. Therefore, the observed associations should not be interpreted as causal or as evidence of acupuncture-specific efficacy, but rather as exploratory findings that inform hypothesis generation for future prospective studies with prespecified, standardized treatment protocols and detailed component-level rehabilitation documentation.

Importantly, the absence of a non-acupuncture or sham-acupuncture control group in this study precludes any inference regarding the specific efficacy of acupuncture itself. All patients received concurrent individualized cognitive training, occupational therapy, exercise therapy, physical therapy, and pharmacological management, each of which may independently or synergistically contribute to cognitive recovery. Consequently, the associations identified in this study should be interpreted as reflecting the experience of patients receiving acupuncture within a comprehensive rehabilitation package, rather than as evidence of acupuncture-specific effects. The clinical implication of our findings is therefore not that “acupuncture works,” but that among patients already receiving such multimodal rehabilitation, certain treatment parameters that were not randomized at baseline—particularly early initiation, higher frequency, and longer duration—showed exploratory associations with more favorable outcomes in this cohort. These findings should not be interpreted as evidence of acupuncture-specific efficacy nor as causal treatment effects.

## 5 Study limitations

The study design is a single-center retrospective cohort study. Although multivariate regression analysis controlled for multiple confounding variables, potential residual confounding and selection bias cannot be completely excluded. In particular, 12 patients who interrupted acupuncture treatment during hospitalization were excluded because the post-treatment MoCA assessment could not be completed. These patients may differ systematically from those who completed the full treatment course, and their exclusion may therefore introduce attrition/selection bias and limit the generalizability of our findings. Since no follow-up MoCA data were available for these patients, a sensitivity analysis including them was not feasible. This limitation should be considered when interpreting the observed associations. Furthermore, while we excluded patients with a pre-existing diagnosis of dementia, we did not have access to standardized, prospectively collected measures of pre-morbid cognitive function. Consequently, undiagnosed pre-stroke mild cognitive impairment may have been present in some patients and could represent an important source of baseline heterogeneity. This limitation may have influenced the observed cognitive trajectories and should be carefully considered when interpreting our results. Furthermore, the assessment of patients’ cognitive function relied on recorded data from the electronic medical record system, and the consistency among different assessors was not subject to standardized calibration, which may introduce certain measurement bias. Although the acupuncture treatment protocol was relatively standardized in our department, individual differences in manipulation techniques, control of deqi sensation, and acupoint selection among different acupuncturists still exist in clinical practice, and these variables were not quantitatively analyzed in this study. Furthermore, this study only included patients who received acupuncture treatment during hospitalization, lacking long-term follow-up data after discharge. The long-term maintenance effects of acupuncture treatment and the long-term outcomes of cognitive function recovery remain unclear. Although our analysis showed that length of hospital stay, assessment interval, and concurrent rehabilitation exposure did not differ between groups, residual confounding from unmeasured factors (e.g., home-based rehabilitation after discharge, caregiver support, or baseline motivation) cannot be fully excluded. Consequently, the observed association between longer treatment course and better cognitive outcomes should be interpreted as exploratory, and causality cannot be established from this retrospective design.

Additionally, our variable selection strategy for the multivariable logistic regression model was constrained by the sample size-to-events ratio. With only 78 patients in the non-improvement group, including all clinically relevant covariates would have risked model overfitting. The primary reason for not incorporating length of hospital stay and total rehabilitation hours into the main multivariable model was the limited number of non-improvement events. Since the final model already included 10 candidate variables, the events-per-variable (EPV) ratio was approximately 7.8:1—which is slightly below the conventional 10:1 threshold recommended to avoid model overfitting. Including length of hospital stay and total rehabilitation hours as additional covariates would have further reduced the EPV ratio to approximately 6.5:1, substantially increasing the risk of unstable estimates or spurious associations. We therefore adopted a restricted approach, prioritizing variables with established prognostic value in PSCI literature. While we acknowledge that this is less ideal than a prespecified covariate selection strategy, this modeling decision was necessitated by the limited number of non-improvement events. Future large-scale, prospective studies with sufficient statistical power are needed to validate our findings with more comprehensive covariate adjustment. Furthermore, internal validation was not performed, and this is acknowledged as a limitation. Consequently, the absence of these variables and the lack of internal validation may introduce residual confounding and limit the generalizability of our findings. All results presented in this study should therefore be interpreted as exploratory and hypothesis-generating rather than confirmatory, and external validation in prospective, multicenter studies is needed to verify the robustness of the associations observed. Furthermore, we acknowledge that model performance evaluation was not comprehensive in this exploratory analysis. The linearity-in-the-logit assumption for continuous predictors was assessed using the Box–Tidwell approach, and no evidence of violation was found; however, we did not perform internal validation through bootstrap resampling, and we did not calculate the area under the curve (AUC) for model discrimination. The model was constructed for explanatory (associational) rather than predictive purposes, and no claims regarding its predictive performance are made. These limitations constrain the generalizability of our findings. Therefore, the findings presented should be interpreted with caution, and future prospective studies with larger sample sizes are warranted to conduct comprehensive model diagnostics to confirm the robustness of our observations.

Although our analysis showed that length of hospital stay, total rehabilitation hours, time from stroke onset to baseline assessment, and the interval between baseline and final assessment did not differ significantly between groups, residual confounding from unmeasured factors cannot be fully excluded. Importantly, total rehabilitation hours were aggregated across cognitive training, occupational therapy, exercise therapy, and physical therapy, and our electronic medical record system did not record the duration or intensity of each component separately. Given that cognitive-specific training is the most relevant concurrent intervention for MoCA outcomes, the lack of component-level data represents a significant limitation. Furthermore, the non-significant between-group comparisons do not constitute evidence of no confounding, as unmeasured variables—including patient motivation, family support, early clinical response, and post-discharge rehabilitation adherence—may have influenced both treatment allocation and cognitive outcomes. We therefore caution against attributing the observed associations to any specific component of the multimodal rehabilitation package, including acupuncture.

Furthermore, the use of a 2-point change in MoCA as the grouping criterion is inherently arbitrary and may be influenced by measurement error, practice effects, or regression to the mean. While this threshold was chosen based on published estimates of minimal clinically important differences, it remains an operational definition for exploratory subgrouping rather than a validated clinical endpoint. Therefore, the descriptive group comparisons and the logistic regression results based on this threshold should be interpreted with caution and are not equivalent to demonstrating clinically confirmed cognitive recovery. Another noteworthy observation is that patients in the non-improvement group, despite failing to achieve the predefined ΔMoCA ≥2 threshold, still experienced significant improvements in mood (HAMD/HAMA), sleep quality (PSQI), and activities of daily living (MBI). This suggests that recovery in these domains may follow distinct trajectories from cognitive recovery, and that patients can derive meaningful functional benefits from the rehabilitation program even in the absence of measurable cognitive gains. This finding underscores the multidimensional nature of post-stroke recovery and supports the continued provision of comprehensive rehabilitation to all patients, regardless of their cognitive response to treatment.

## 6 Conclusion

This retrospective, exploratory study, conducted in PSCI patients receiving acupuncture within a multimodal inpatient rehabilitation setting, identified exploratory associations between earlier initiation, higher weekly frequency, longer total treatment duration, and more favorable cognitive outcomes in this cohort, while advanced age, greater neurological severity at admission, and comorbid diabetes mellitus were associated with poorer outcomes. These associations were observed in the absence of a non-acupuncture control group and therefore cannot establish acupuncture-specific efficacy or causality. They should not be used to guide clinical practice or to recommend specific treatment protocols. Instead, these findings serve as hypothesis-generating observations that may inform the design of future prospective, multicenter, randomized controlled trials with appropriate control arms, which are urgently warranted to validate these associations and to determine whether any causal therapeutic effects exist.

## Data Availability

The raw data supporting the conclusions of this article will be made available by the authors, without undue reservation.
